# Eosinophilic gastroenteritis with gastric and small bowel involvement: successful treatment with oral budesonide

**DOI:** 10.1186/1710-1492-8-S1-A6

**Published:** 2012-11-02

**Authors:** Mohammad Alsayegh, Douglas Mack

**Affiliations:** 1 Department of Medicine, Mubarak Al-Kabeer Hospital, Kuwait; 2 Department of Pediatrics, McMaster University, Hamilton, Ontario, Canada

## Introduction

Eosinophilic gastroenteritis (EGE) is an uncommon gastrointestinal disorder. The manifestations of EGE are varied and steroids are the mainstay of treatment. To our knowledge we present the first case of successful treatment of pediatric EGE with both budesonide in oral suspension and enteric-coated budesonide.

## Case report

A 16 year old female with a chronic history of mild abdominal discomfort presented with acute severe abdominal pain. Her absolute eosinophil count was elevated as was her total IgE. Skin testing, serology and other bloodwork were negative. Computed tomography scan of abdomen and pelvis showed diffuse thickening of the duodenum. Histopathological studies of gastric and duodenal biopsies revealed heavy eosinophilic infiltration. She had improvement on prednisone however the patient's symptoms relapsed after discontinuing. Her symptoms were then controlled on a daily dose of 11 mg of oral budesonide in two different formulations. 2 mg of daily budesonide was given in the form of sweetened oral suspension and 9 mg of daily budesonide was given in the form of enteric coated (EC) budesonide oral tablets The patient reported significant improvement of symptoms within 5 days of starting the budesonide combination. The patient continued budesonide treatment for one month. She has been asymptomatic to 13 months following treatment with follow up endoscopy revealing full resolution of eosinophilic infiltration.

## Conclusion

Oral budesonide can be useful in the treatment of EGE. We suggest that careful selection of budesonide formulations for EGE treatment is important to ensure correct drug delivery to the affected gut.

